# Does Increasing Active Warm-Up Duration Affect Afternoon Short-Term Maximal Performance during Ramadan?

**DOI:** 10.1371/journal.pone.0116809

**Published:** 2015-02-03

**Authors:** Hana Baklouti, Asma Aloui, Hamdi Chtourou, Walid Briki, Anis Chaouachi, Nizar Souissi

**Affiliations:** 1 Research Laboratory “Sport Performance Optimization”, National Center of Medicine and Sciences in Sport, Tunis, Tunisia; 2 High Institute of Sport and Physical Education of Gafsa, University of Gafsa, Gafsa, Tunisia; 3 High Institute of Sport and Physical Education of Sfax, University of Sfax, Sfax, Tunisia; 4 University of French West Indies and Guyana, ACTES Laboratory, Pointe-à-Pitre, Guadeloupe, France; Texas A&M University, UNITED STATES

## Abstract

**Aim:**

The purpose of this study was to examine the effect of active warm-up duration on short-term maximal performance assessed during Ramadan in the afternoon.

**Methods:**

Twelve healthy active men took part in the study. The experimental design consisted of four test sessions conducted at 5 p.m., before and during Ramadan, either with a 5-minute or a 15-minute warm-up. The warm-up consisted in pedaling at 50% of the power output obtained at the last stage of a submaximal multistage cycling test. During each session, the subjects performed two vertical jump tests (squat jump and counter movement jump) for measurement of vertical jump height followed by a 30-second Wingate test for measurement of peak and mean power. Oral temperature was recorded at rest and after warming-up. Moreover, ratings of perceived exertion were obtained immediately after the Wingate test.

**Results:**

Oral temperature was higher before Ramadan than during Ramadan at rest, and was higher after the 15-minute warm-up than the 5-minute warm-up both before and during Ramadan. In addition, vertical jump heights were not significantly different between the two warm-up conditions before and during Ramadan, and were lower during Ramadan than before Ramadan after both warm-up conditions. Peak and mean power were not significantly different between the two warm-up durations before Ramadan, but were significantly higher after the 5-minute warm-up than the 15-minute warm-up during Ramadan. Moreover, peak and mean power were lower during Ramadan than before Ramadan after both warm-up conditions. Furthermore, ratings of perceived exertion were higher after the 15-minute warm-up than the 5-minute warm-up only during Ramadan.

**Conclusion:**

The prolonged active warm-up has no effect on vertical jump height but impairs anaerobic power assessed during Ramadan in the afternoon.

## Introduction

Ramadan is the ninth month of the lunar Islamic calendar during which Muslims are requested to abstain from eating, drinking, and other behaviors from dawn to sunset. This fasting duration depends on the season during which Ramadan occurs and the geographical location, and could be longer than 18 hours per day in the summer of temperate regions. Ramadan is characterized by several behavioral changes especially related to the sleep-wake cycle and eating schedule, which may affect sport performance [1]. Research dealing with the effect of Ramadan fasting on sport performance presented inconsistent results. Some studies reported slight decrements or no significant effect [2,3], while others found significant impairments in sport performance during Ramadan [4–6]—sprint performance [7], vertical jump height [8], muscle power [4,6,9], and performance during endurance exercises [7,8,10,11] were lower during Ramadan compared to non-Ramadan periods.

The mechanisms potentially responsible for these inconsistencies are not well established yet, even if authors speculated that they could be attributed to a diversity of factors [12,13] such as the moment of experimental sessions. Concerning this factor, most studies reported significant reductions in short-term maximal performance assessed during Ramadan compared to the out-of-Ramadan control period when test sessions were conducted in the afternoon but not in the morning [4,5,9,14]. For instance, using a 30-second Wingate test, Chtourou et al. [4] reported a lower peak (PP) and mean (MP) power during Ramadan (i.e., at the second and the fourth weeks of Ramadan) than before Ramadan in the afternoon without any significant difference between the two testing periods in the morning. In most studies, the decrease in short-term maximal performance during Ramadan in the afternoon was associated with a decrease in core temperature [5,9], suggesting that the decrease in core temperature could be responsible for decrements in exercise performance during Ramadan.

To increase core temperature, the most common method is warming-up. In this context, Souissi et al. [15] showed that increasing warm-up duration induces an enhancement in short-term maximal performance in the morning without any significant effect in the evening. However, Racinais et al. [16] observed that a 12-minute active warm-up improves performance in both times-of-day. Moreover, Taylor et al. [17] reported that the effect of a prolonged warm-up in the afternoon for peak and mean power was unclear due to the variety of individual responses.

In the authors’ opinion, prolonging the warm-up period during Ramadan could accentuate the hypohydration induced by daytime fasting, which may impair sport performance. Therefore, the aim of the present study was to assess whether the increase in active warm-up duration would affect vertical jump height, PP, and MP measured during Ramadan in the afternoon.

## Materials and Methods

### Participants

Twelve healthy active men (age 21.11 ± 2.75 years, body mass 70.3 ± 9.1 kg, height 1.73 ± 0.06 m) gave written consent to participate in the study after receiving a meticulous explanation of the protocol. The study was approved by the Clinical Research Ethics Committee of the National Center of Medicine and Sciences in Sport of Tunis. The elapsed time between dawn and sunset was from 03:51 a.m. to 07:20 p.m. at the beginning and from 04:20 a.m. to 06:49 p.m. at the end of Ramadan. During this period, the participants took their last meal (i.e., *“suhur”*) approximately at ~01:00 a.m. and fasted from then until sunset.

### Study Design

Two weeks before the actual measurements, the subjects were familiarized with vertical jumping and high-intensity cycling on cycle-ergometer to minimize the learning effect. The experimental design consisted of two testing phases: two weeks before Ramadan (BR) and the end of the second week of Ramadan (R2). During each period, the subjects randomly performed two test sessions at the same time-of-day (05:00 p.m.) with a recovery period > 36 hours in-between: one session with a 5-minute warm-up and another with a 15-minute warm-up. Five minutes after warming-up, the subjects performed a squat jump (SJ) test, a counter movement jump (CMJ) test, and then a 30-second Wingate test. Ratings of perceived exertion (RPE) were recorded after the Wingate test.

Oral temperature was measured at rest and after each warm-up with a clinical thermometer inserted sublingually for 3 minutes.

To determine the warm-up intensity, the subjects performed an incremental maximal test on a cycle-ergometer before the beginning of the experiment. The procedure was as follows: after 3 minutes of cycling at 50 W, the intensity of the exercise increased by 25 W every 2 minutes until exhaustion. The power corresponding to 50% of the power output recorded in the last stage of the incremental test was chosen as the warm-up intensity [15].

Throughout the study, the participants were requested to avoid strenuous activities during the 24 hours preceding each test session.

### Squat Jump and Counter Movement Jump Tests

The SJ consists in a maximal vertical jump from a flexed position, while the CMJ is a leg flexion from the standing position immediately followed by a maximal vertical jump. Both tests were performed with the hands on the hips. The vertical jump tests were conducted using a vertical jump meter which records the jump height. Three trials were completed for each test, with a 2-minute rest period between jumps, and the best ones (highest jumps) were retained for further analysis.

### Wingate Test

The Wingate test was conducted on a friction-loaded cycle ergometer interfaced with a microcomputer. This test requires pedaling for 30 s at maximal speed against a constant force related to body mass (0.087 kg ∙ kg^−1^ body mass) [18]. The test began from a rolling start against minimal resistance. When a constant pedal rate of 60 rpm was achieved, the test resistance was applied and the subjects were instructed to pedal as fast as they could during 30 s. During the test, they had to remain seated and were strongly encouraged to reach the maximal pedaling rate as quickly as possible.

PP was measured as the highest mechanical power that is elicited in the test and MP was the average power that is sustained throughout the 30-second period.

### Ratings of Perceived Exertion

Perceived exertion is an indicator of the degree of physical strain [19]. The RPE scale used in the present study presents 15 points ranging from 6 (very very light) to 20 (very very hard) [20]. The higher the RPE score, the higher the rating of perceived exertion.

### Statistical Analysis

Data are presented as mean values ± SD. The Shapiro-Wilk *W*-test for normality revealed that the data were normally distributed. PP, MP, SJ height, CMJ height, and RPE values were analyzed using a two-way analysis of variance (ANOVA) with repeated measures (2 [Period] × 2 [Warm-up]). Oral temperature data were analyzed using a three-way ANOVA with repeated measures (2 [Period] × 2 [Warm-up] × 2 [Measure]). When appropriate, significant differences between means were tested using the Fisher’s Least Significant Difference (LSD) test. Effect sizes were determined using partial eta squared (η_p_^2^) to estimate the magnitude of effects. Statistical significance was established at *p* < 0.05.

## Results

### Oral Temperature

**Figure 1:**
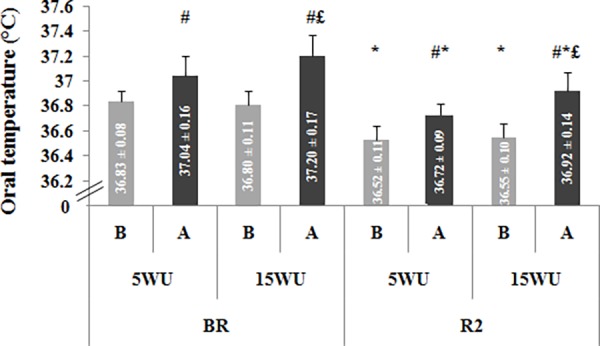
Oral temperature (°C) (mean ± SD, n = 12) recorded before (BR) and during (R2) Ramadan, before (B) and after (A) the 5-minute (5WU) and the 15-minute (15WU) warm-up. #: significant difference in comparison with before warm-up. *: significant difference in comparison with BR. £: significant difference in comparison with the 5-minute warm-up.

Oral temperature values measured at rest and after each warm-up, during the two testing phases, are displayed in Fig. 1.

The period (F = 141.78, η_p_^2^ = 0.93, *p* < 0.001), warm-up (F = 6.70, η_p_^2^ = 0.38, *p* < 0.05), and measure (F = 201.93, η_p_^2^ = 0.95, *p* < 0.001) effects were significant. However, the period × warm-up × measure interaction was not significant (F = 0.006, η_p_^2^ = 0.001, *p* > 0.05).

The Fisher’s LSD test showed that resting values of oral temperature were significantly higher BR than during R2 (*p* < 0.001). Oral temperature increased from before to after the 5-minute (*p* < 0.01) and the 15-minute (*p* < 0.001) warm-up in the two testing periods. Moreover, oral temperature was significantly higher after the 15-minute warm-up than the 5-minute warm-up BR (*p* < 0.05) and during R2 (*p* < 0.01).

### Vertical Jump Tests

**Figure 2:**
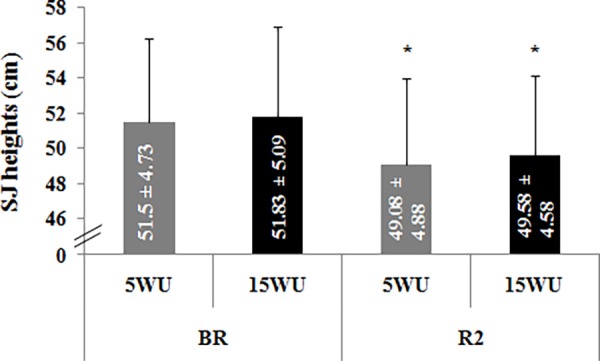
Squat jump heights (cm) (mean ± SD, n = 12) measured after the 5-minute (5WU) and the 15-minute (15WU) warm-up, before (BR) and during (R2) Ramadan. *: significant difference in comparison with BR.

**-Squat Jump Height**. SJ heights measured in the four test sessions are shown in Fig. 2.

The statistical analysis showed significant main effects of period (F = 35.63, η_p_^2^ = 0.77, *p* < 0.001) and warm-up (F = 5.18, η_p_^2^ = 0.32, *p* < 0.05). However, the period × warm-up interaction was not significant (F = 0.04, η_p_^2^ = 0.01, *p* > 0.05).

The LSD test revealed that, in the two testing phases, there was no significant difference between the SJ heights measured after the two warm-up periods. Moreover, SJ heights were significantly lower during R2 than BR after the 5-minute (*p* < 0.001) and the 15-minute (*p* < 0.01) warm-up.

**Figure 3:**
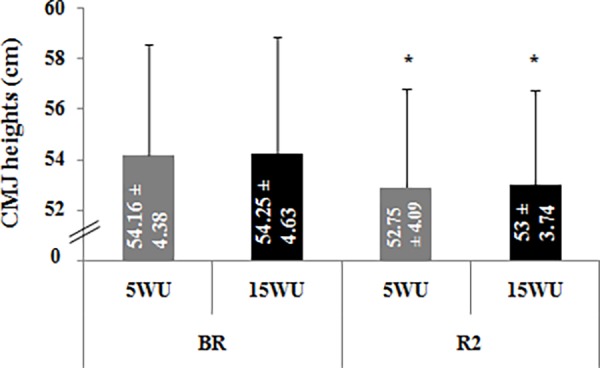
Counter movement jump heights (cm) (mean ± SD, n = 12) measured after the 5-minute (5WU) and the 15-minute (15WU) warm-up, before (BR) and during (R2) Ramadan. *: significant difference in comparison with BR.

**-Counter Movement Jump Height**. CMJ heights measured in the four test sessions are presented in Fig. 3.

A significant main effect of period was found (F = 20.11, η_p_^2^ = 0.65, *p* < 0.001). However, the warm-up effect (F = 0.42, η_p_^2^ = 0.04, *p* > 0.05) and the period × warm-up interaction (F = 0.05, η_p_^2^ = 0.01, *p* > 0.05) were not significant.

The LSD test revealed that, in the two testing periods, CMJ heights were not significantly different between the two warm-up durations. Furthermore, CMJ heights were significantly lower during R2 than BR after the 5-minute and the 15-minute warm-up (*p* < 0.05).

### Wingate Test

**Figure 4:**
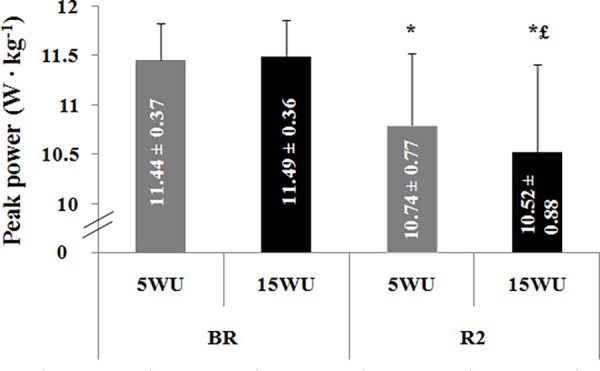
Peak power (W ∙ kg^−1^) (mean ± SD, n = 12) determined during the 30-second Wingate test, after the 5-minute (5WU) and the 15-minute (15WU) warm-up, before (BR) and during (R2) Ramadan. *: significant difference in comparison with BR. £: significant difference in comparison with the 5-minute warm-up.

**-Peak Power**. PP determined during the 30-second Wingate test in the four test sessions are displayed in Fig. 4.

The statistical analysis showed a significant main effect of period (F = 25.63, η_p_^2^ = 0.70, *p* < 0.001). However, the warm-up effect (F = 2.07, η_p_^2^ = 0.16, *p* > 0.05) and the period × warm-up interaction (F = 4.24, η_p_^2^ = 0.28, *p* > 0.05) were not significant.

The LSD test showed that, BR, PP was not significantly different between the two warm-up durations. However, during R2, PP was higher after the 5-minute warm-up than the 15-minute warm-up (*p* < 0.05). Moreover, PP was lower during R2 than BR after the 5-minute and the 15-minute warm-up (*p* < 0.001).

**Figure 5:**
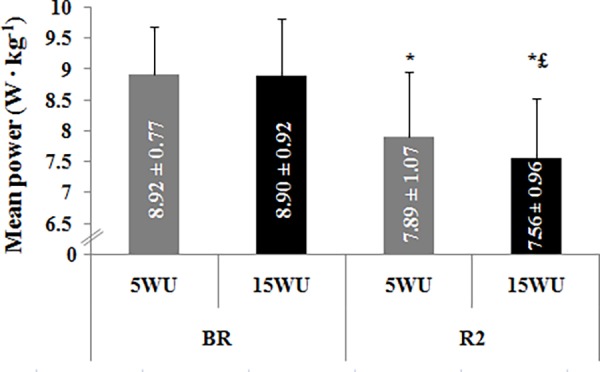
Mean power (W ∙ kg^−1^) (mean ± SD, n = 12) determined during the 30-second Wingate test, after the 5-minute (5WU) and the 15-minute (15WU) warm-up, before (BR) and during (R2) Ramadan. *: significant difference in comparison with BR. £: significant difference in comparison with the 5-minute warm-up.

**-Mean Power**. MP determined during the 30-second Wingate test in the four test sessions are presented in Fig. 5.

Significant main effects of period (F = 71.48, η_p_^2^ = 0.87, *p* < 0.001) and warm-up (F = 5.48, η_p_^2^ = 0.33, *p* < 0.05) and a significant period × warm-up interaction (F = 6.09, η_p_^2^ = 0.36, *p* < 0.05) were found.

The Fisher’s test showed that, BR, MP was not significantly different between the two warm-up durations. However, during R2, MP was higher after the 5-minute warm-up than the 15-minute warm-up (*p* < 0.01). Furthermore, MP was higher BR than during R2 after both warm-up periods (*p* < 0.001).

### Ratings of Perceived Exertion

**Figure 6:**
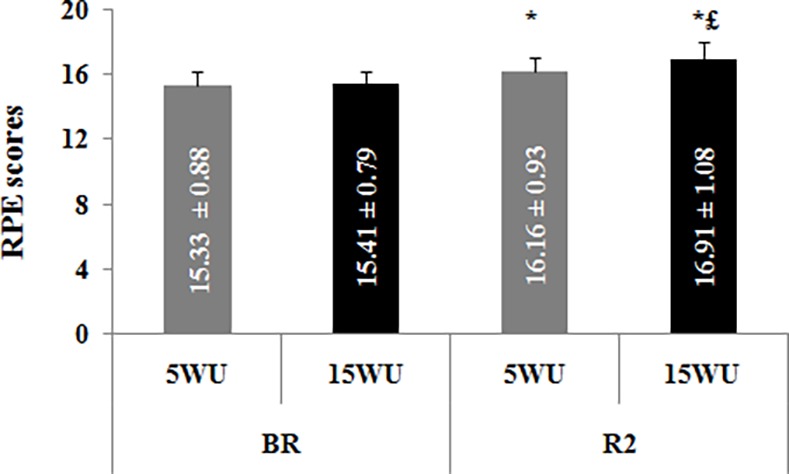
RPE scores (mean ± SD, n = 12) obtained following the 30-second Wingate test, after the 5-minute (5WU) and the 15-minute (15WU) warm-up, before (BR) and during (R2) Ramadan. *: significant difference in comparison with BR. £: significant difference in comparison with the 5-minute warm-up.

RPE scores obtained in the four test sessions are shown in Fig. 6.

The period effect was significant (F = 36.61, η_p_^2^ = 0.67, *p* < 0.001). However, the warm-up effect (F = 3.01, η_p_^2^ = 0.38, *p* > 0.05) and the period × warm-up interaction (F = 2.57, η_p_^2^ = 0.16, *p* > 0.05) were not significant.

BR, there was no significant difference between the two warm-up durations for RPE scores. However, during R2, RPE scores were significantly higher after the 15-minute warm-up than the 5-minute warm-up (*p* < 0.05). Moreover, RPE scores were significantly higher during R2 than BR after both warm-up periods (*p* < 0.001).

## Discussion

The present study revealed that, BR, PP and MP were not significantly different between the two warm-up durations; however, during R2, these performance measures were higher after the 5-minute warm-up than the 15-minute warm-up. Moreover, PP and MP were lower during R2 than BR after both warm-up periods. In addition, SJ and CMJ heights were not significantly different between the two warm-up durations in the two testing phases, and were lower during R2 than BR after both warm-up periods.

Our results confirm those of Souissi et al. [15] and showed that oral temperature increased after both warm-up periods, with no significant difference between the two warm-up durations for short-term maximal performance assessed BR in the afternoon. These findings could be explained by the existence of a “ceiling” after which power does not increase even though central temperature does [21–23]. However, our findings run counter to those of Racinais et al. [16] who found that maximal cycling sprints’ maximal power assessed in the afternoon was higher after the longer warm-up. The authors speculated that the beneficial effects of an active warm-up would not be dependent only on the temperature increase [24] because decreased muscle and joint stiffness would be caused by the breaking of the stable bonds between actin and myosin filaments [25].

The findings of the present study confirm that short-term maximal performance can be affected during the fasting month in the afternoon [5,11,14,26]. For instance, in Tunisian male soccer players, PP and MP in a 30-second Wingate test were lower during the second and the fourth weeks of Ramadan than BR in the afternoon [4]. It has been demonstrated that the 30-second Wingate test requires a high level of motivation to observe maximal performance [4,27]. Therefore, the reduced performance observed during Ramadan could be due to a lower motivation level compared to before Ramadan [28]. Moreover, Zerguini et al. [7] and Chtourou et al. [11,29] suggested that decrements in the players’ mood and motivation would be possible factors responsible for the reduction in sport performance during Ramadan. Furthermore, as previously shown [4,11], we observed higher RPE scores during R2 compared to BR. The higher RPE scores suggest that Ramadan fasting may result in an increased level of fatigue. Also, the higher RPE scores could be assigned to negative mood. The decline in mood and mental activity may induce a reduction in physical performance [30,31].

During R2, we found that PP and MP were lower after the 15-minute warm-up than the 5-minute warm-up. This result could be explained by the hypohydration induced by the prolonged warm-up. Indeed, a long warm-up could result in a higher sweat loss rate and then may accentuate the hypohydration induced by daytime fasting. Acute hypohydration may affect sport performance [32], eye-hand coordination [33], and cognitive functions [33–35], and leads to an increased perception of fatigue [36–38]. Furthermore, Judelson et al. [39] indicated that body water loss has various effects on neuromuscular function and short-term power. Indeed, strength, power, and high intensity endurance are limited by hypohydration [39]. The published evidence suggests that there is a likelihood of a reduction in strength if hypohydration is induced as a result of prolonged food and fluid restriction [40,41].

Moreover, the lower power observed after the 15-minute warm-up as compared to the 5-minute warm-up could be explained by the fatigue induced by the longer warm-up that is added to the fatigue caused by ~16 hours of fasting. Accordingly, the present study demonstrated that RPE scores were higher after the 15-minute warm-up than the 5-minute warm-up during R2.

SJ and CMJ heights were not significantly different between the two warm-up durations during R2. Likewise, Judelson et al. [40] found that hypohydration up to 4.8% body mass has a little demonstrable effect on vertical jump height, peak lower-body power assessed via jump squat, and peak lower-body force assessed via isometric back squat. They suggested that if hypohydration fails to reduce muscle force or power, vertical jump height should increase as total body water decreases because the jumper must move less mass.

## Conclusions

The present study showed that a prolonged active warm-up has no effect on vertical jump height but impairs anaerobic power assessed during Ramadan in the afternoon. Therefore, there is no need to prolong the warm-up period before short-term maximal exercises performed during Ramadan in the afternoon.
